# Chemosterilant Potential of Insect Growth Regulators for Management of *Bactrocera cucurbitae* (Diptera: Tephritidae)

**DOI:** 10.3390/insects16020137

**Published:** 2025-01-31

**Authors:** Iqra Kainat, Shafqat Saeed, Muhammad Asif Farooq, Wafa A. H. Alkherb, Asim Abbasi, Farrukh Baig, Umer Liaqat, Fawad Zafar Ahmad Khan, Muhammad Irfan Akram, Muhammad Hasnain, Nazih Y. Rebouh

**Affiliations:** 1 Institute of Plant Protection, Muhammad Nawaz Shareef University of Agriculture, Multan 60000, Pakistan; 2 Department of Biology, College of Science, Qassim University, P.O. Box 6666, Buraidah 51452, Saudi Arabia; 3 Department of Entomology, University of Agriculture, Faisalabad 38040, Pakistan; asimuaf95@gmail.com; 4 Department of Zoology, Wildlife and Fisheries, University of Agriculture, Faisalabad 38040, Pakistan; 5 Department of Outreach and Continuing Education, Muhammad Nawaz Shareef University of Agriculture Multan, Multan 60000, Pakistan; 6 Department of Entomology, Faculty of Agriculture and Environment, The Islamia University of Bahawalpur, Bahawalpur 63100, Pakistan; 7 Department of Environmental Management, Institute of Environmental Engineering, RUDN University, 6 Miklukho-Maklaya St., Moscow 117198, Russia

**Keywords:** chemosterilant bait, environmental management, IPM, melon fruit fly, tephritid, vegetables

## Abstract

The melon fruit fly, *Bactrocera cucurbitae*, is an important insect pest responsible for significant yield losses in vegetables. Conventional control methods, like pesticide applications, have certain limitations, including environmental contamination. This study evaluated the chemosterilant potential of five new insect growth regulators (IGRs)—Pyriproxyfen; Novaluron; Lufenuron; Buprofezin; and Flubendiamide—at concentrations ranging from 50 to 300 ppm in a 5 mL diet against *B. cucurbitae*. Laboratory trials identified Lufenuron as the most effective IGR, which reduced fecundity by 68.4% and adult emergence by 70.97% at a 300 ppm concentration. Other IGRs, Pyriproxyfen; Novaluron; Buprofezin; and Flubendiamide, also significantly reduced fecundity and adult emergence at higher concentrations. Based on laboratory performance, Lufenuron was selected for field trials using six bait traps per 0.4 hectares. The field trials demonstrated a higher number of *B. cucurbitae* adult captures in Lufenuron-based bait traps compared to the control and reductions in crop damage, with fruit fly damage decreasing by 7.01% in August and 4.25% in September. This study highlights the potential of chemosterilant baited traps as a promising approach for integrated fruit fly management programs in cucurbitaceous vegetables.

## Simple Summary

The melon fruit fly (*Bactrocera cucurbitae*) is a significant insect pest causing substantial yield losses in vegetable crops. Traditional control methods, including pesticide applications, face numerous challenges such as cost-effectiveness, environmental contamination, and emergence of resistant insect biotypes. The current study investigates the chemosterilant efficacy of five new insect growth regulators (IGRs), Pyriproxyfen, Novaluron, Lufenuron, Buprofezin, and Flubendiamide, at different concentrations against *B. cucurbitae*. Laboratory trials identified Lufenuron as the most effective IGR, reducing fecundity by 68.4% and adult emergence by 70.97% at the highest tested concentration. Other IGRs also significantly affected fecundity and emergence at higher concentrations. Lufenuron was selected for field trials using chemosterilant bait traps, which captured more adults than control treatment and reduced fruit fly damage by 7.01% in August and 4.25% in September. This research demonstrates the potential of chemosterilant bait traps as an environmentally friendly strategy for the integrated management of *B. cucurbitae* in cucurbitaceous vegetables.

## 1. Introduction

Melon fruit fly, *Bactrocera cucurbitae* (Coquillett) (Diptera: Tephritidae), is an important pest of the cucurbitaceous vegetables, including bitter gourd (*Momordica charantia* L.), snap melon (*Cucumis melo* L. var. *momordica*), muskmelon (*Cucumis melo* L.), sponge gourd (*Luffa aegyptiaca* Mill.), and snake gourd (*Trichosanthes cucumerina* L. var. *anguina*) [1,2,3,4]. Since its first report in 1913, this pest has now spread to more than 40 countries across the globe [5,6]. The infestation associated with melon fruit fly begins when female flies lay eggs 2–5 mm deep inside the flesh of ripe fruit [7]. The immatures emerge from the oviposited eggs and start feeding, which leads to early fruit decay [8]. Moreover, the commercial value of the infested fruit has been compromised due to economic sanctions, such as quarantines and loss of agricultural capital [9]. Fruit damage caused by *B. cucurbitae* can range from 30% to 100% in the case of severe infestation [1,10,11]. Female fruit flies have a broad host range for oviposition and remain active most of the year, making them a persistent threat to vegetable production systems [1].

Several control methods have been deployed in field conditions to manage *B. cucurbitae*; however, insecticides are still preferred by the farming community due to their quick action mechanisms. Traditional insecticides used to suppress the *B. cucurbitae* population include organophosphates, pyrethroids, avermectins, and spinosyns [12]. However, the frequent application of these pesticides often leads to environmental and health issues, and therefore there is a growing amount of research focusing more on safer insect control tactics. Insect growth regulators (IGRs), which hinder the growth and development of insects, are emerging as a promising management tool [13]. Prominent IGR groups include chitin synthesis inhibitors, juvenile hormone mimics, ecdysone agonists, and molting disruptors [13,14]. Notably, IGRs have minimal effects on non-target organisms, making them an environment friendly option as compared to conventional insecticides [14,15]. Previously, Lufenuron, Novaluron, Buprofezin, Diflubenzuron, and Triflumuron acting as chitin synthesis inhibitors [16,17,18,19]; Pyriproxyfen, Fenoxycarb, and Hydroprene acting as juvenile hormone antagonists [19,20]; Methoprene and Methoxyfenozide acting as molting hormone mimics [17,21]; Flubendiamide acting on receptors in the muscles [20]; and Flufenoxuron inhibiting growth and development [21] have been used against fruit flies and many other insect pests.

Adult flies are the most prominent stage of *Bactrocera* species observed in the field, where they feed on sugar-rich exudations. Incorporating the specific concentrations of IGRs in food bait induces sterility in adult flies [22,23]. Previous studies have demonstrated the sterilization effects of IGRs against different fruit fly species. For example, Azadirachtin (inhibit reproduction and produce sterility in insects), Lufenuron, and Triflumuron have been effective against Mediterranean fruit fly *Ceratitis capitata* (Wiedemann) (Diptera: Tephritidae) [24,25,26]. Similarly, Azadirachtin, Cyromazine, Flufenoxuron, Lufenuron, and Pyriproxyfen were found to be effective against olive fly, *Bactrocera oleae* (Rossi) (Diptera: Tephritidae) [23]. Additionally, Lufenuron, Cyromazine, Pyriproxyfen, Azadirachtin, and Tebufenozide have been used against spotted-wing drosophila *Drosophila suzukii* (Matsumura) (Diptera: Drosophilidae) [23]. Lufenuron was found to be effective against *Bactrocera dorsalis* (Hendel) (Diptera: Tephritidae) [24]. Moreover, sterilization effects of Pyriproxyfen, Novaluron, Lufenuron, and Buprofezin have also been reported against *Bactrocera zonata* (Saunders) (Diptera: Tephritidae) [27].

Research examining the effects of IGRs on *B. cucurbitae* remains limited. Therefore, the aim of the current study was to evaluate the chemosterilant effects of several IGRs with different modes of action on *B. cucurbitae*. Among the IGRs tested, Lufenuron and Flubendiamide were included for their chitin synthesis inhibitor properties, with Lufenuron also exhibiting ovicidal properties [28,29]. Novaluron, another chitin synthesis inhibitor, was used for its ability to reduces egg viability and prevent oviposition in female fruit flies [30,31]. We also included Buprofezin, which delays chitin formation [32], and Pyriproxyfen, which acts as a juvenile hormone mimic, i.e., it disrupts the progression of immature stages and also effects reproductive potential [15]. Following the selection of IGRs, we conducted a series of experiments. Initially, the effects of these IGRs on the fecundity of *B. cucurbitae* were evaluated. Next, the IGR that demonstrated the highest sterilizing effect in preliminary trials was further tested for its sex-specific sterility effect. Finally, field trials were performed using IGR-treated baits to monitor their impact on fruit fly damage.

## 2. Materials and Methods

### 2.1. Insect Collection and Rearing

Infested vegetables, including bitter gourd (*Momordica charantia* L.), luffa gourd (*Luffa aegyptiaca* Mill.), snake gourd (*Trichosanthes cucumerina* L.), and round gourd (*Benincasa fistulosa* (Stocks), were collected from a field located near Muhammad Nawaz Shareef University of Agriculture, Multan, Pakistan, during the summer season of 2021–2022. The infested vegetables were brought to the fruit fly laboratory and placed on an approximately 2 cm thick layer of sterilized sand inside a disposable plastic container (30 cm × 20 cm × 10 cm). This container was then placed inside a transparent rearing cage (60 cm × 40 cm× 40 cm) until adult emergence. The rearing cage possessed a wired mesh for the constant air flow inside the cage. The cages were placed in a controlled environment room (CER) under the adjusted environmental conditions: photoperiod of 12:12 (day and light), 65-75% RH (relative humidity), and a temperature range of 25–27 °C. The temperature and humidity were maintained using an air conditioner coupled with a humidifier. A similar set of laboratory conditions were maintained for the behavior trials. After adult emergence, the specimens were identified using a standard taxonomic key [33]. The adults of *B. cucurbitae* were then transferred to fresh rearing cages with water and a mixture of brewer’s yeast and sugar (3:1 *w*/*w*) as adult food.

### 2.2. Larval Diet Preparation

An artificial larval diet was prepared by mixing the following ingredients into a consistent paste: 250 g of luffa gourd, one teaspoon of honey, 50 g of sugar, 50 g of egg yolk, 1 g of yeast, and three to four drops of Vitamin B complex syrup. Then, the diet was poured into plastic trays (15 cm × 10 cm × 3 cm) wrapped with cling wrap to prevent desiccation and stored at 4 °C in a refrigerator. Larval diet cups were prepared using 10 mL of the larval diet inside 20 mL clear plastic cups, which were used in rearing. The fruit fly oviposition cups were prepared by cutting a small piece of filter paper (1 cm × 2 cm), followed by dipping it into the larval diet, and placing it inside the cup with four equidistant holes to facilitate egg laying. The oviposition cups were placed inside the rearing cages and left undisturbed for 24 h. The filter papers with eggs were then transferred to larval diet cups. Fully grown larvae coming out of the cups were placed over a 1–2 cm thick layer of sterilized sand in a plastic container for pupation. Once the larvae pupated, the container with sand and pupae was transferred to another cage.

### 2.3. Chemicals

Five commercial formulations of IGRs were used in this study: Lufenuron^®^ [5% *w*/*v* (emulsifiable concentrate: EC) Lufenuron] (Jaffer Brothers, Pakistan), Priority^®^ [10.8% *w*/*v* (EC) Pyriproxyfen] (Kanzo Ag., Pakistan), Uniron^®^ [10% *w*/*v* (EC) Novaluron] (ICI Pakistan Ltd.), Trofezon^®^ [25% *w*/*v* (wettable powder: WP) Buprofezin] (Tara Groups, Pakistan), and Belt^®^ [48% *w*/*v* (suspension concentrate: SC) Flubendiamide] (Bayer Crop Sciences). Stock solutions were prepared by cleaning glass beakers with ethanol (70%). After cleaning, the glass beakers were rinsed with distilled water and dried by inverting them on paper towels placed on a laboratory shelf. Distilled water was used to prepare all the stock and working solutions. The stock solution of each insecticide was prepared using one liter of distilled water with the following concentrations: Lufenuron: 1 mL, Pyriproxyfen: 2.5 mL, Novaluron: 3 mL, Buprofezin: 5 g, Flubendiamide: 0.5 mL. Six different test concentrations (50, 100, 150, 200, 250, and 300 ppm) were made by serial dilution of each stock solution [34]. Distilled water without any chemical served as the control treatment.

### 2.4. Effect of IGRs on B. cucurbitae Fecundity and Adult Emergence

Ten pairs of freshly emerged adults of *B. cucurbitae* flies were placed in separate plastic cages (6 cm × 4 cm × 4 cm) with mesh netting on two sides for aeration. Inside each cage, 5 mL of adult diet (described above) treated with test concentrations of insecticides and an insecticide-free diet (control) were provided. The insecticide concentrations were mixed with 5 mL of acetone and allowed to evaporate before offering the diet to the flies. After 24 h, the treated diet was replaced with a normal (insecticide-free) diet. The test concentrations of each pesticide and a control were replicated five times using a completely randomized design (CRD). Eggs from each cage were collected by using oviposition cups (method has already been described above) daily from the first to the last day of oviposition. Fecundity was then determined by dividing the total number of eggs laid by female fruit flies in each cage. Eggs from each treatment were transferred to new rearing cages, and the previously mentioned rearing protocol was followed until adult emergence.

### 2.5. Effect of Lufenuron on Sex-Specific Sterility

Based on the previous experiment, Lufenuron at 300 ppm (5 mL of diet), which resulted in the lowest fecundity and adult emergence, was selected to further investigate its effects on the sterility of *B. cucurbitae* adults. The same experimental procedure was followed, but only one IGR (Lufenuron) at one concentration (300 ppm) was tested against the control. After feeding ten-day-old male and female adults separately on the treated diet for 24 h, the following pairs were set up: untreated females (UFs) × treated males (TMs), treated females (TFs) × untreated males (UMs), treated females (TFs) × treated males (TMs), and untreated females (UFs) × untreated males (UMs). Each cross included ten pairs of *B. cucurbitae* per cage, with five replications for each treatment.

### 2.6. Field Testing of Lufenuron-Treated Bait

The chemosterilant IGRs were used with an attractive bait. They contained protein hydrolysate, ammonium acetate, jaggery, putrescine, and trimethylamine. Detailed methodology regarding bait formulation was provided in our previous study [27]. The field trials were conducted from July to September 2022. The first field trial was performed to estimate the attractiveness of the bait, while the second one aimed to evaluate the chemosterilant potential of the bait combined with IGRs. Experiments were performed in two luffa gourd fields (0.4 hectare). To avoid the mixing of local fruit fly populations, a 500 m distance between the luffa gourd fields was maintained. The luffa gourd field was not sprayed with pesticides, and routine management operations, like weeding and irrigation, were performed on regular intervals. In the attraction experiment, six Steiner traps were deployed in the field (0.4 hectare). Two 20 mm holes were made on each side of the traps to allow for fruit fly entry. A cotton swab soaked with 25–30 mL of bait solution was placed inside the traps, and water-only swabs were kept as the control treatment. For the chemosterility experiment, a disposable plastic bottle of 2L volume was used. The bottle was painted yellow and two rectangular holes (7.5 × 5 cm) were made using a paper cutter to allow for fruit fly entry [27]. In both experiments, the baits were placed 3–4 feet above ground level and changed on a weekly basis.

### 2.7. Fruit Infestation Assessment

A sample of 100 fruits were randomly assessed for fruit fly infestation. The infestation was determined by observing the oviposition puncture marks on the fruits. After this initial observation, ten fruits were dissected to confirm the presence of larvae. Infestation severity was assessed by counting healthy and infested vegetables. Percent damage was calculated from a sample size of 100 fruits per acre, with the number of infested fruits divided by the total number of fruits observed and multiplied by 100.

### 2.8. Statistical Analysis

Data on fecundity and adult emergence were analyzed using an analysis of variance (ANOVA) to determine the significance of the means. Differences between the means of the IGR concentrations were determined using the least significant difference (LSD) test at the 5% significance level. To determine the effect of sex-specific sterility, data of crosses from different treatment groups, i.e., treated males vs. treated females, untreated males vs. treated females, treated males vs. untreated females, and untreated males vs. untreated females, were analyzed using a general linear model. The model was constructed using fecundity or adult emergence as dependent variables, cross between male and female as the fixed factor, and days as the random factor. Interaction between crosses and days was determined to assess the effect of different days on the fecundity of female *B. cucurbitae* in each cross. To compare the efficacy of bait traps compared to the control (water only) traps, a paired t-test was used. Data were analyzed using SPSS Statistics software package (version 27).

## 3. Results

### 3.1. Effect of IGRs on Fecundity and Adult Emergence

The oral toxicity of insect growth regulators (IGRs) on *B. cucurbitae* significantly reduced the fecundity (eggs per female) and adult emergence (number of adults emerging from pupae) when exposed to varying concentrations of IGRs (50, 100, 150, 200, 250, and 300 ppm/5mL of diet).

#### 3.1.1. Effect of IGR Concentrations on Fecundity

**Figure 1:**
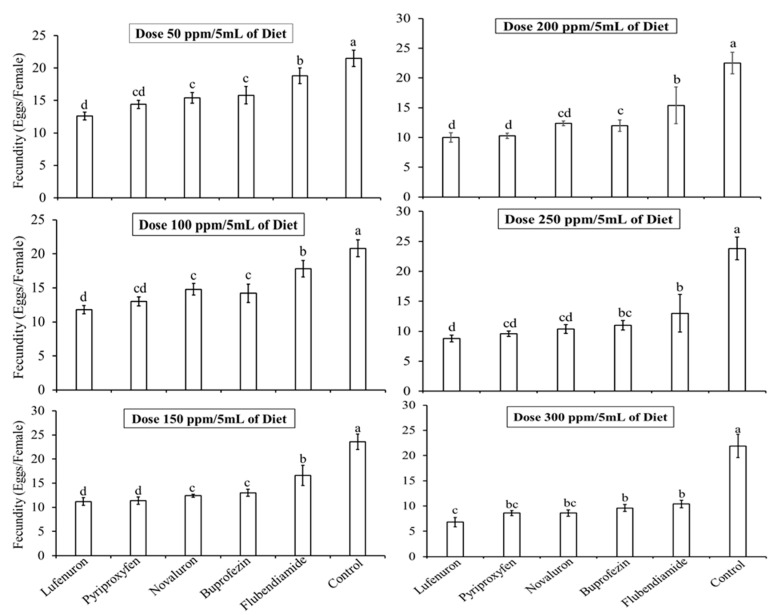
Effect of different concentrations of insect growth regulators (IGRs) on the fecundity (mean ± SE) of *Bactrocera cucurbitae*. Graphs presents the mean number of eggs laid (fecundity) by *B. cucurbitae* females when fed with different IGRs mixed with baits. Bars sharing identical letters show no significant difference in fecundity at the 5% probability level, as determined by statistical analysis.

The egg laying potential of *B. cucurbitae* was significantly reduced (*F* = 197.18, df = 103, *p* < 0.05) when exposed to the diet with six different concentrations of IGRs. Each of the five IGR levels demonstrated varying degrees of sterility. Overall, the Lufenuron proved to be the most effective IGR in reducing the fecundity of *B. cucurbitae* females across all the tested concentrations, although the fecundity reduction due to Lufenuron was not significantly different from Pyriproxyfen. The fecundity data for adult *B. cucurbitae* on the lowest concentration, i.e., 50 ppm/5 mL diet (Figure 1) showed variability across the treatments. As compared to the control treatment (21.5 ± 1.25 eggs/female), females fed on the Lufenuron mixed diet laid the lowest number of eggs (12.6 ± 0.61 eggs/female), followed by Pyriproxyfen (14.4 ± 0.65), Novaluron (15.4 ± 0.83), Buprofezin (15.8 ± 1.34), and Flubendiamide (18.8 ± 1.21). At the highest concentration, i.e., 300 ppm/5 mL of diet, the maximum reduction in fecundity of *B. cucurbitae* adults was observed. Females fed on the Lufenuron-treated diet laid the lowest mean number of eggs (6.80 ± 0.92) compared to the Pyriproxyfen (8.6 ± 0.52), Novaluron (8.6 ± 0.61), Buprofezin (9.6 ± 0.67), and Flubendiamide (10.4 ± 0.75) diets and the control (21.9 ± 2.30).

#### 3.1.2. Effect of IGR Concentrations on Adult Emergence

**Figure 2:**
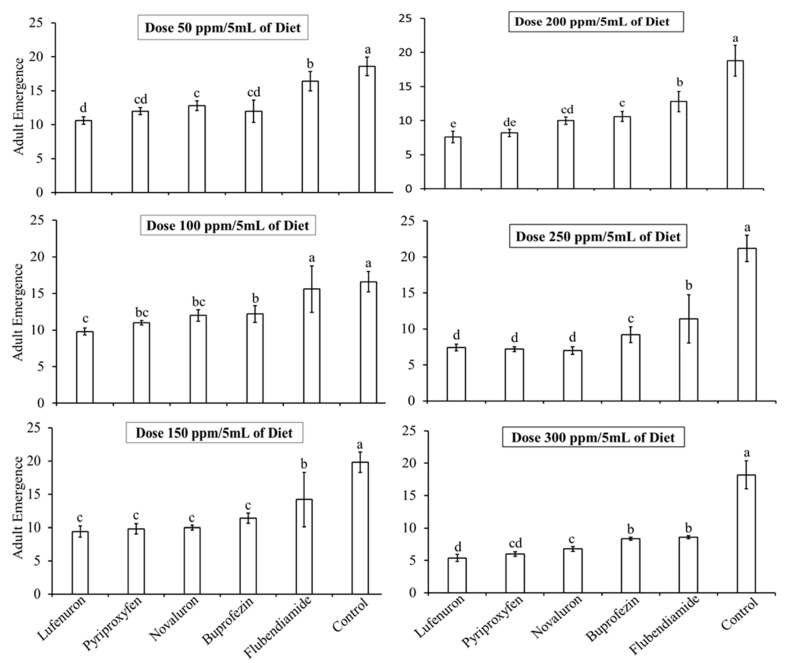
Effect of different concentrations of insect growth regulators (IGRs) on the emergence of adult *Bactrocera cucurbitae* (mean ± SE). The graph shows the mean emergence rate of adult *B. cucurbitae* from pupae, when adults were fed with different IGRs mixed with baits. Bars sharing the same letters indicate no significant difference in adult emergence at the 5% probability level, as determined by statistical analysis.

All IGR treatments significantly reduced the emergence of adult *B. cucurbitae* from pupae (*F* = 195.32, df = 102, *p* < 0.05). The lowest adult emergence was observed at higher concentrations (300 ppm/5 mL), while higher emergence rates were recorded at lower concentrations (50 ppm/5 mL). At 50 ppm/5 mL, the Lufenuron-treated diet resulted in the lowest mean of adult emergence (10.6 ± 0.56), whereas the Pyriproxyfen, Buprofezin, Novaluron, and Flubendiamide treatments resulted in 12.00 ± 0.53, 12.8 ± 0.71, 12 ± 1.64, and 16.4 ± 1.41 mean adult emergence, respectively, compared to the control (18.6 ± 1.35) (Figure 2). At 300 ppm/5 mL, the Lufenuron-treated diet resulted in the lowest mean of adult emergence (5.4 ± 0.52), whereas the Pyriproxyfen, Buprofezin, Novaluron, and Flubendiamide treatments resulted in 6 ± 0.34, 6.8 ± 0.37, 8.4 ± 0.23, and 8.6 ± 0.24 mean adult emergence, respectively, compared to the control (18.20 ± 2.16). Thus, Lufenuron showed the lowest fecundity and adult emergence rates, although differences were not significant compared to the other IGRs used in this study.

### 3.2. Effect of Lufenuron on Sex-Specific Sterility

#### 3.2.1. Effect of Lufenuron-Induced Sex-Specific Sterility on Egg Deposition

**Figure 3:**
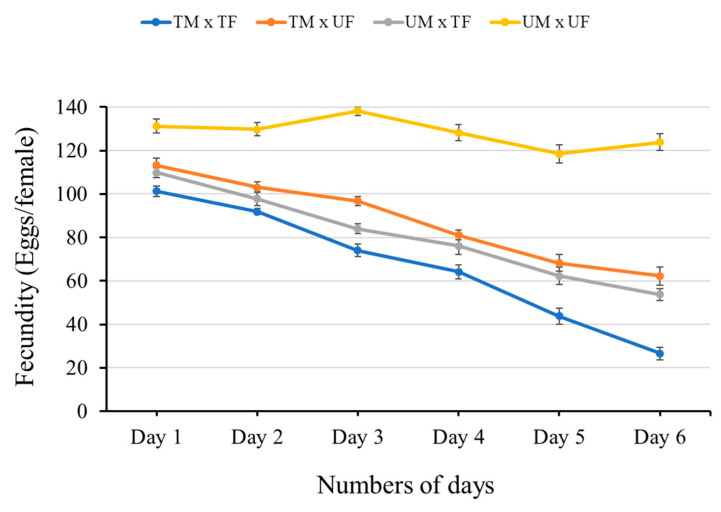
Effect of Lufenuron-induced sex-specific sterility on fecundity (mean ± SE) in *Bactrocera cucurbitae* across different days. The graph shows the mean number of eggs laid (fecundity) by *B. cucurbitae* females after different combinations of mating pairs exposed with or without chemosterilant-based diet. Error bars represent the standard error (SE) of the mean.

A significant reduction in oviposition was observed when 300 ppm of Lufenuron per 5 mL of diet was fed to *B. cucurbitae* adults. Egg deposition by the female *B. cucurbitae* in all crosses was significantly reduced (Table 1 and Figure 3) compared to the control group (untreated male vs. untreated female). The maximum reduction (74%) in egg deposition was observed in the pair where both male and female flies were fed the Lufenuron-treated diet (day 1 = 101.23 ± 2.33; day 6 = 26.55 ± 2.78). Meanwhile, no significant differences were observed in the mean number of eggs between the TM × UF and UM × TF pairs. In the pairs, where only one sex (either male or female) was fed the Lufenuron-treated diet, egg reduction was no more than 50% from day 1 to day 6. The pair, which was not fed the Lufenuron-treated diet, laid an almost consistent number of eggs from day 1 to day 6 (Table 1 and Figure 3).

#### 3.2.2. Effect of Lufenuron-Induced Sex-Specific Sterility on Adult Emergence

**Figure 4:**
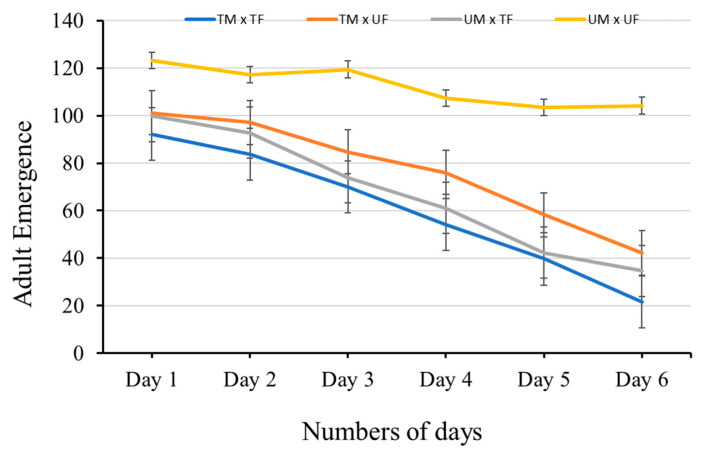
Effect of Lufenuron-induced sex-specific sterility on adult emergence (mean ± SE) in *Bactrocera cucurbitae* across different days. The graph illustrates the mean emergence rate of adult *B. cucurbitae* from pupae, which developed from the eggs of females, which mated in different combinations of mating pairs exposed with or without chemosterilant-based diet. Error bars represent the standard error (SE) of the mean.

The effects of the Lufenuron-treated diet on adult emergence were almost similar to egg deposition. Adult emergence in all crosses was significantly reduced (Table 2 and Figure 4) compared to the control group (untreated male vs. untreated female). The maximum reduction (77%) in adult emergence was observed in the pair where both male and female flies were fed the Lufenuron-treated diet (day 1 = 92.23 ± 3.45; day 6 = 21.55 ± 2.09), while no significant differences were observed in mean number of adults that emerged from the TM × UF and UM × TF pairs. In the pairs, where only one sex (either male or female) was fed the Lufenuron-treated diet, adult emergence was no more than 65% from day 1 to day 6. The pair that was not fed the Lufenuron-treated diet laid an almost consistent number of eggs from day 1 to day 6 (Table 2 and Figure 4).

### 3.3. Field Testing of Bait Traps for Adult Captures and Damage Assessment

**Figure 5:**
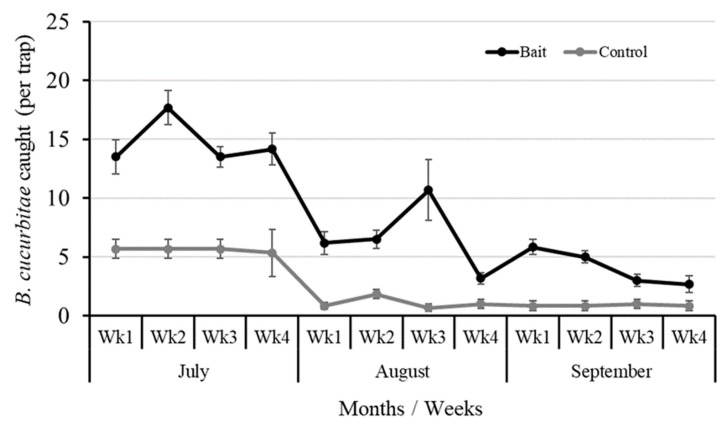
Number of *B. cucurbitae* caught per trap using Lufenuron-based bait versus control. The graph compares the mean number of *B. cucurbitae* individuals caught per trap using Lufenuron-based bait against the control traps during different weeks of July, August, and September. Error bars represent the standard error (SE) of the mean.

**Figure 6:**
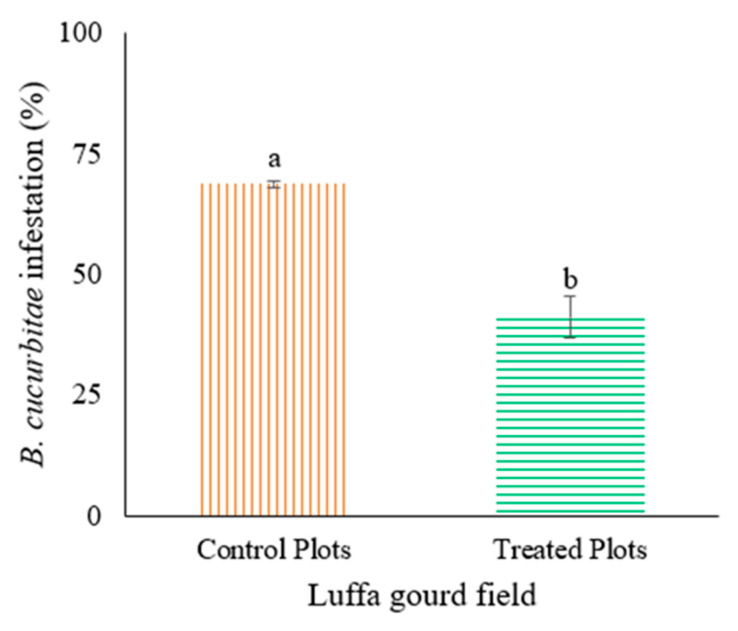
Percent *B. cucurbitae* damage in luffa gourd field treated with Lufenuron baits compared to control field. Bars sharing the different letters indicate significant difference in *B. cucurbitae* infestation at the 5% probability level.

**Figure 7:**
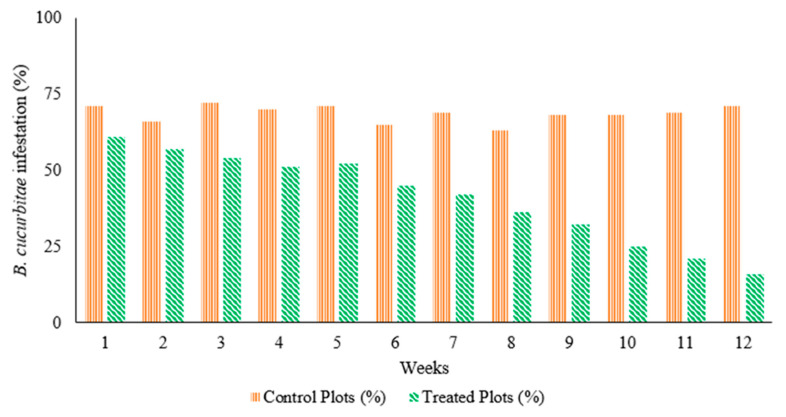
Percent damage by *B. cucurbitae* in luffa gourd field treated with Lufenuron baits compared to control field across different weeks of this study.

While testing the efficacy of the Lufenuron-treated bait traps in capturing adult *B. cucurbitae* under field conditions, we found that a significantly higher number of flies were captured (t_70_ = 11.25, *p =* <0.001) in bait traps (8.56 ± 0.67 per trap) compared to the control (water only) traps (2.54 ± 0.33 per trap) (Figure 5). Generally, the population decreased from July to the end of August, with the highest population recorded for July (58.1), followed by August (33.5) and September (23) in 2022 (Figure 5). The field experiment on luffa gourd infestation caused by *B. cucurbitae* demonstrated that the installation of bait traps significantly reduced the infestation level (t_11_ = −6.43; *p* = < 0.01) compared to the water traps (control) (Figure 6). This reduction was consistent throughout the experimental period. On the first week, the infestation in the luffa gourd field with bait traps was 13% lower compared to the control field (Figure 7). Over time, the bait traps proved to be more effective where this difference was increased to almost 50% by the end of the experimental duration.

## 4. Discussion

Our study provided evidence of fruit fly management in luffa gourd using IGR-based chemosterilant baits. It is always a challenge to manage the melon fruit fly populations in the field. On the other hand, consumers always demand fruit-fly-infestation-free agricultural produce [35]. Pesticide sprays dominate the fruit fly pest control programs, but effective management still remains a challenge [36]. The use of chemosterilants has been demonstrated to be effective for managing fruit flies, as demonstrated in the current study. Insect growth regulators have potential to reduce fecundity and adult emergence. Furthermore, IGRs are well suited for fruit fly management due to their ecological compatibility and target specificity [36,37]. However, different growth regulators vary in their sterility-inducing potential, probably due to differences in their modes of action.

The current study showed that the IGRs, when mixed with attractive bait, induced sterility in fruit fly populations. Chemosterilants induce sterility in insects because of their ill effects on the metabolism of nucleic acids, which ultimately hinders the synthesis of proteins associated with the development of the ovaries or testes [38,39,40]. Lufenuron was the most effective IGR in reducing adult emergence and fecundity in *B. cucurbitae*. Navarro-Llopis et al. [41] also reported the role of Lufenuron in causing sterility in *C. capitata*, which is consistent with our results. A study on *Drosophila suzukii* also showed that Lufenuron, Cyromazine, and Pyriproxyfen caused a reduction in fertility, egg laying potential, and egg hatch [42]. Moreover, another study also supports the role of IGRs in reducing developmental rates, fecundity, and adult emergence from pupae [43]. Similar effects of Lufenuron on the hatching ability of *C. capitata* eggs have also been reported by Casana-Giner et al. [44] and Nisar et al. [36].

Fecundity and adult emergence were reduced in crosses between males and females treated with Lufenuron. These results are similar to those reported by Moya et al. [43], who found that the mating of treated males and females resulted in sterile offsprings in *Anastrepha ludens* (Loew) (Diptera: Tephritidae). Lufenuron affects chitin synthesis in insects by inhibiting enzyme chitin synthase. This failure to produce chitin leads to mortality, especially in immature stages, like larvae, and in freshly emerged adults. It also reduces egg development and fecundity, as shown in boll weevils [45].

Decisions regarding control measures and their effectiveness are based on trapping data. Significant differences were observed in the number of *B. cucurbitae* throughout the season (*p* < 0.05). The population density of *B. cucurbitae* was highest in July and later declined during August and September mainly due to unfavorable environmental conditions. There is a close relationship between attractants (catch/traps) and the months of captured *B. cucurbitae*. Epsky and Heath [46] noted that capturing systems are sufficient and effective, capturing both female and male insects. The type or design of the trap influences the adult capture of several insects [47]. Pheromone traps, tested against various insect pests [48], have been widely used for monitoring purposes related to managing insect pest populations.

Chemosterilants offer notable advantages due to their simplicity and cost-effectiveness, and they do not require genetic manipulation or regulatory approvals before their deployment. Moreover, chemosterilants were also preferred due to their strong selectivity and unique mode of action, against which the chances of resistance development are very low [49,50]. The sterile insect technique (SIT) [51,52] has been successfully used to eradicate different fruit fly species in different countries, for example *A. ludens* in Mexico [53], *Ceratitis capitata* in the Dominican Republic [54], and *B. tryoni* in Australia [55]. However, due to its high costs and the difficulty of applying irradiations to sterilize insects in developing countries, cheaper and more cost-effective methods are needed. Thus, chemosterilants provide an alternative to costly sterilization methods in developing countries. Lufenuron (an IGR) has been characterized as a chemosterilant for a wide range of insects, including tephritid pests [37,56], reducing adult fertility and fecundity [57].

## 5. Conclusions

The current study assessed melon fruit fly behavior to develop effective management models and explore new control options, i.e., IGR-based chemosterilant bait. Chemosterilants based on IGRs offer a viable solution for managing *B. cucurbitae* infestations without harming non-target organisms and the environment. This technique has an advantage over mass trapping due to the prolonged attractive effect of protein-based material, supported by visual and olfactory cues. We recommend chemosterilant baiting throughout the year so as to reduce the fruit fly populations in a specific field and ultimately in a given area. This management option will help improve agricultural production and farmer income.

## Figures and Tables

**Table 1:** General linear model showing effect of crosses across different days on fecundity of *B. cucurbitae* along with interaction between crosses and days.

Source	*F*	Df	*P*
Cross	453.18	3	<0.001
Days	8.85	5	<0.001
Cross × Days	1882.81	15	<0.001

**Table 2:** General linear model showing effect of crosses across different days on adult emergence of *B. cucurbitae* along with interaction between crosses and days.

Source	*F*	df	*p*
Cross	320.17	3	<0.001
Days	8.39	5	<0.001
Cross × Days	61.47	15	<0.001

## Data Availability

The data used and analyzed during this research work can be requested from corresponding authors on reasonable requests.
